# Biopharmaceutical profiling of anti-infective sanggenons from *Morus alba* root bark for inhalation administration

**DOI:** 10.1016/j.ijpx.2024.100272

**Published:** 2024-08-05

**Authors:** Jacqueline Schwarzinger, Sigrid Adelsberger, Karin Ortmayr, Sarah Luise Stellnberger, Ammar Tahir, Gabriela Hädrich, Verena Pichler, Judith M. Rollinger, Ulrike Grienke, Lea Ann Dailey

**Affiliations:** a Division of Pharmaceutical Technology and Biopharmaceutics, Department of Pharmaceutical Sciences, Faculty of Life Sciences, University of Vienna, Josef-Holaubek-Platz 2, 1090 Vienna, Austria; b Vienna Doctoral School of Pharmaceutical, Nutritional and Sport Sciences, University of Vienna, Josef-Holaubek-Platz 2, 1090 Vienna, Austria; c Division of Pharmacognosy, Department of Pharmaceutical Sciences, Faculty of Life Sciences, University of Vienna, Josef-Holaubek-Platz 2, 1090 Vienna, Austria; d Division of Pharmaceutical Chemistry, Department of Pharmaceutical Sciences, Faculty of Life Sciences, University of Vienna, Josef-Holaubek-Platz 2, 1090 Vienna, Austria

**Keywords:** *Morus alba*, Moraceae, Inhalation, Biopharmaceutical profiling, Pharmacokinetic synergy, Permeability

## Abstract

Mulberry Diels-Alder-type adducts (MDAAs), isolated from *Morus alba* root bark, exhibit dual activity against viral and bacterial pathogens but show sobering efficacy following oral administration. Inhalation administration may overcome issues with oral bioavailability and improve efficacy for the treatment of respiratory infections. To assess the suitability of MDAAs for inhalation administration, physicochemical (e.g. pH, pK_a_, logP, pH-dependent solubility) and biopharmaceutical (epithelial cytotoxicity, permeability, and uptake) properties of two bioactive MDAA stereoisomers sanggenon C (SGC) and sanggenon D (SGD) were evaluated as isolated natural compounds and within parent extracts (MA21, MA60). Despite their structural similarity, SGD exhibited a 10-fold higher solubility than SGC across pH 1.2–7.4, with slight increases at neutral pH. Both compounds were more soluble in isolated form than in the parent extracts. The more lipophilic SGC was found to be more cytotoxic when compared to SGD, indicating a better cellular penetration, which was confirmed by uptake studies. Nonetheless, SGC and SGD exhibited no measurable permeability across intact Calu-3 monolayers, highlighting their potential for increased lung retention and improved local anti-infective activity following inhalation administration. Results suggest that SGC and SGD in isolated form, rather than as extracts, are promising candidates for pulmonary drug delivery to treat lung infections.

## Highlights

• Mulberry Diels-Alder-type adducts (MDAAs) are investigated for pulmonary delivery.
• Biopharmaceutical profiling demonstrated promising pharmacokinetic properties.
• Stereoisomeric MDAAs differ in pH dependent solubility and cytotoxicity.
• Tested MDAAs showed highly suitable characteristics for lung delivery systems.
• Isolated compounds have more favourable properties than herbal parent extracts.

## Graphical abstract

**Figure:**
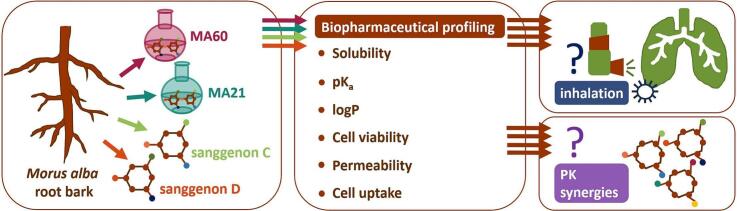

## 1 Introduction

Poor or undesired biopharmaceutical properties are, next to adverse effects and lack of efficacy, one of the main reasons for lead candidate failure during non-clinical and clinical drug development (Prentis et al., 1988; Waring et al., 2015). Currently, biopharmaceutical profiling studies are often performed in the lead candidate optimization phase for synthetic small molecule drug candidates (Kerns and Di, 2003; Venkatesh and Lipper, 2000) but not routinely investigated for promising compounds isolated from natural sources, such as herbal extracts (Blume and Schug, 2000). Nonetheless, there is a growing body of literature documenting the biopharmaceutical behaviour of natural product-derived compounds in isolated versus extract form. The term “pharmacokinetic synergy” (PK synergy) has recently been used to describe the observation that important physicochemical and biopharmaceutical properties of natural product-derived active pharmaceutical ingredients (APIs), such as solubility and cell permeability, can differ substantially when the key constituents are in isolated form or in their corresponding herbal extract (Li et al., 2011; Machado et al., 2020; Zhao et al., 2020). Many studies found an increased solubility of the analyte in the presence of coexisting compounds (Jürgenliemk and Nahrstedt, 2003; Keung et al., 1996; Kornievskaya et al., 2007; Ma et al., 2016; Xu et al., 2007). Interestingly, PK synergies between constituents of natural product extracts have been reported to increase or decrease the permeability of plant constituents (Ta et al., 2023; Zheng et al., 2012; Zheng et al., 2015; Zhu et al., 2013). In some cases, PK synergies of the extract have been shown to improve therapeutic efficacy compared to the purified bioactive compound (Hengjumrut et al., 2018; Joo et al., 2010; Song et al., 2007; Weathers et al., 2011; Wu et al., 2009; Zhang et al., 2014; Zhang et al., 2015). Thus, understanding PK synergies is an important step towards designing a suitable target product profile (TPP) for therapeutic agents derived from natural products. This type of investigation addresses the question of whether the extract or isolated bioactive agent would exhibit favourable properties in the drug product.

**Fig. 1:**
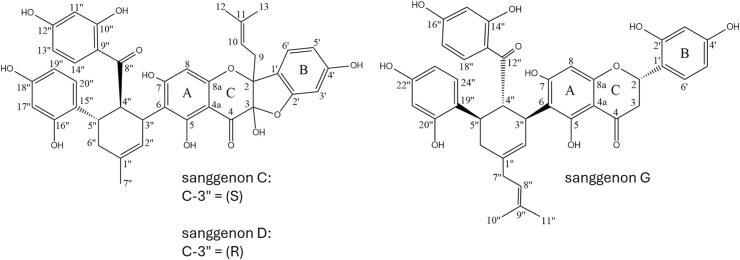
Structures of stereoisomers sanggenon C (SGC) and sanggenon D (SGD), both prenylated in position C-2, and sanggenon G (SGG) with an isoprenyl unit in position C-1″.

The white mulberry tree, i.e. *Morus alba* L. (Moraceae), has a long history of traditional medicinal use in Asian countries (Singh et al., 2013). Especially root bark preparations have been reported as oral treatment options for various lung inflammatory disorders such as acute and chronic bronchitis due to their antimicrobial and anti-inflammatory activity (Batiha et al., 2023; Chan et al., 2016; Lim et al., 2013). Mulberry Diels-Alder-type adducts (MDAAs) such as sanggenon C (SGC), sanggenon D (SGD), and sanggenon G (SGG), are prenylated flavonoids formed via [4 + 2]-cycloaddition between chalcones and dehydroprenylphenols (Luo et al., 2021, Luo et al., 2022) (Fig. 1) with promising pharmacodynamic activity as anti-infectives. Recent in vitro studies show that MDAAs combat the lethal synergism of viruses (influenza A virus, SARS-CoV-2) and bacteria (pneumococci) (Grienke et al., 2016; Langeder et al., 2023a; Wasilewicz et al., 2023).

However, a major drawback is their low in vivo bioavailability and low plasma/tissue concentration after oral application (Langeder et al., 2023b). Lipinski's rule of five characterizes the properties of a substance, such as molecular weight (< 500 Da), hydrogen bond donors (< 5) and acceptors (< 10), as well as the logP value (< 5), to determine its “drug-likeness” and predict its potential for good oral bioavailability (Lipinski et al., 1997). The poor oral bioavailability of SGC and SGD could be due to the complex molecular structure of the sanggenons, which does not adhere to Lipinski's rule of five because of their high molecular weight (708.7 g/mol) and the presence of eight hydrogen bond donors and 12 hydrogen bond acceptors. The lack of oral bioavailability may be overcome by local drug delivery to the lung. Since respiratory infections are one of the primary target disease areas for MDAAs, the option of developing an inhalation product instead of an oral formulation is an attractive solution to circumvent this dilemma and at the same time benefit from a targeted application.

Marchand and Couet have extensively investigated the aerosol administration of antibiotics for pulmonary infections, to understand molecular properties favourable for lung retention and therefore improved local efficacy. They observed that molecules characterized by a large, hydrophilic structure and low permeability across lung epithelial monolayers exhibit favourable pulmonary pharmacokinetics, with high lung concentrations and low systemic exposure. The investigated antibiotics showed the following P_app_ values: tobramycin (P_app_ < 0.05 × 10^−6^ cm/s), aztreonam (P_app_ 0.07 ± 0.02 × 10^−6^ cm/s), and colistin (P_app_ 0.04 ± 0.02 × 10^−6^ cm/s), which are defined as low P_app_ values (Galindo Bedor et al., 2016; Gontijo et al., 2014a; Gontijo et al., 2014b; Marchand et al., 2010, Marchand et al., 2015, Marchand et al., 2016, Marchand et al., 2018). In comparison, metoprolol, which represents the border between high and low permeability, has a P_app_ of 10.3 ± 0.0 × 10^−6^ cm/s in Calu-3 cells (Bosquillon et al., 2017).

Taking these observations into consideration, we hypothesize that sanggenons may also exhibit prolonged retention in the lungs. However, no physicochemical and biopharmaceutical data on sanggenons as a class of molecules have been published. To address this lack of information, two MDAA-containing extracts (MA21 and MA60) and their isolated major MDAA stereoisomers (SGC and SGD) were selected as representative compounds for the evaluation of (i) pH-dependent solubility, (ii) pK_a_, (iii) lipophilicity (logP), (iv) cell viability, (v) epithelial permeability and (vi) cell uptake. Due to their structural characteristics, we hypothesize that sanggenons might exhibit low permeability characteristics and therefore show a potential for prolonged lung retention. The study provides relevant data for the possible development of an inhaled drug product based on MDAA-containing extracts or purified bioactive compounds (i.e. sanggenons) and acts as a general template for biopharmaceutical profiling of natural product-derived APIs. Such molecules can often show a surprising degree of chemical complexity compared to standard synthetic small-molecule APIs and can therefore present a substantial challenge for subsequent formulation.

## 2 Materials and methods

### 2.1 Materials

Fetal bovine serum (FBS), phosphate-buffered saline (PBS), Hanks' balanced salt solution (HBSS), fluorescein sodium salt, metoprolol tartrate and dimethyl sulfoxide (DMSO, purity 99.8%, CAS: 67–68-05) were obtained from Sigma-Aldrich (St. Louis, USA). Dulbecco's modified Eagle medium/nutrient mixture F-12 (DMEM/F-12), GlutaMAX™ supplement, penicillin-streptomycin (10,000 U/mL) solution, trypsin-EDTA (0.25%) and thiazolyl blue tetrazolium bromide (MTT) were purchased from Thermo Fisher Scientific (Waltham, USA). Ammonia solution Rotipuran® (NH_3_ + H_2_O), was purchased from Carl Roth GmbH + Co. KG (Karlsruhe, Germany). LC-MS grade methanol (MeOH), was purchased from VWR Chemicals (Radnor, USA). Double-distilled deionized water (dd H_2_O) for preparation of solubility and permeability samples was produced by a Milli-Q-Plus ultrapure water device (Millipore Corporation, Bedford, MA, USA) and by an Arium® Pro Ultrapure Lab Water System from Sartorius (Göttingen, Germany). Reagent grade tert-butyl methyl ether (MTBE), was purchased from Sigma-Aldrich (St. Louis, USA). Formic acid (FA) p.a., used as an additive for mobile phases, was purchased from Chem-lab nv (Zedelgem, Belgium).

### 2.2 Extracts and pure compounds

One batch of two respective *M. alba* root bark extracts, MA21 and MA60, (Langeder et al., 2023a) were used for this study. MA21 is a hydroethanolic extract (extracted at room temperature with 60 vol% ethanol) containing 1.0% of SGC and 1.1% of SGD, with a total sanggenon content of 2.6%. MA60 was produced by pressurised liquid extraction using *n*-hexane for defatting and isopropanol-petroleum ether (2:1) for extraction to obtain a high content of sanggenons in the final extract. MA60 contains 6.9% of SGC and 10.7% of SGD with an overall total sanggenon content of 29.0%. Both MDAAs, SGC and SGD (purity >98%), were previously isolated from MA60 as described in Langeder et al., 2023a. For quantitative analysis of the permeability assay, SGG was used as an internal standard. This compound, with a purity of 98% according to UPLC-ELSD detection, was also previously isolated and analysed (Langeder et al., 2023a).

### 2.3 pH-dependent solubility profile

The thermodynamic solubility was evaluated with an adapted miniaturized shake-flask method in a pH-dependent manner (Glomme et al., 2005; Veseli et al., 2020). Weighed masses (SGC, SGD: 0.15 mg; MA21: 14.2 mg; MA60: 1.4 mg) were selected to exceed the estimated solubility values determined in preliminary studies by a factor of five, to ensure that saturation conditions were reached. The masses of the extracts were then chosen to contain the same quantity of sanggenons as the purified compounds to improve comparability. MDAA samples were first prepared as stock solutions in methanol. The desired concentrations were then pipetted into new vials, and methanol was evaporated to obtain the required quantities. A volume of 100 μL of the following buffers was added to the sample: HCl buffer (pH 1.2), acetate buffer (pH 4.5), and phosphate buffer (pH 6.8 and 7.4). After five minutes of sonication, the suspensions were incubated under shaking conditions (250 rpm at 24 ± 0.24 °C) for 24 h (Incubator hood TH15 + Compact Shaker KS 15 A control from Edmund Bühler GmbH, Bodelshausen, Germany). Separation of dissolved and non-dissolved compounds was achieved via centrifugation at 6708*g* for 20 min (MIKRO 200/200R, Andreas Hettich GmbH & Co. KG, Tuttlingen, Germany). Supernatants were removed and stored at −20 °C until measurement. Shortly before the measurement, 80 μL of the thawed supernatant samples were centrifuged and neutralised with 0.1% aqueous ammonia, if necessary, to adjust the pH. A 3-cycle MTBE-based extraction protocol using 3 × 750 μL MTBE was then implemented. Quality control (QC) evaluations included low, middle, and high-concentration samples from the calibration curve range. Blanks were prepared by applying the same extraction protocol to the respective buffer systems. Quantitative analysis was performed using an Acquity UPLC H-class system by Waters equipped with an Acquity BEH Phenyl column (2.1 × 100 mm, 1.7 μm) and a PDA detector. Detailed information about the preparation of samples and blanks, QC samples and QC blanks, as well as information about method validation and quantitation using UPLC-PDA can be found in the electronic supplementary information (ESI).

### 2.4 pK_a_ of sanggenons C and D

Experimental pK_a_ (−log_10_ of the acid dissociation equilibrium constant) values of SGC and SGD were evaluated using the UV-metric pK_a_ measurement method of the SiriusT3 automated titrator instrument and the corresponding software SiriusT3Control and SiriusT3Refine (Pion Inc., Billerica, USA). The SiriusT3 apparatus uses a deuterium lamp to determine the UV/Vis absorbance spectrum of the test solutions at each pH point. The standard aqueous UV-metric pK_a_ measurement protocol was conducted according to manufacturer instructions. Briefly, a 10 mM stock solution of SGC and SGD was prepared in DMSO (*n* = 5 replicate samples) and 5 μL was added to a 4 mL Sirius T3 flat bottom tube. This was mixed with 25 μL phosphate buffer (14.7 mM K_2_HPO_4_ and 0.15 M KCl in MilliQ water) and diluted with 1.5 mL ionic strength-adjusted water (ISA water ≡ 0.15 M KCl in in MilliQ water). The subsequent acid/base titration and measurement of UV absorbance was automatically performed by the SiriusT3 at 25.0 ± 0.5 °C. The pH titration was performed in triplicate titrations between pH 1 and 13 by adding acid (0.5 M HCl) and base (0.5 M KOH) under an argon atmosphere. Turbidity was monitored by a turbidity sensor to ensure the absence of precipitation of the test compounds during the experiment, since accurate pK_a_ measurements are limited to fully dissolved substances. In case turbidity occurred, individual titrations were not used for further analysis, resulting in a total of 11 analysable titrations across the 5 replicate samples for both SGC and SGD.

### 2.5 logP of sanggenons C and D

LogP (partition coefficient) evaluation was performed via SiriusT3. The potentiometric logP measurement relies on assessing the partition profile of the test substance within a two-phase water-octanol solvent system. Changes in the relative volume ratio of octanol to water lead to shifts in the apparent pK_a_, resulting from the partitioning between non-ionised and ionised forms. LogP values were then estimated based on these assessments. SGC and SGD (SGC: 0.91 mg, SGD: 1.00 mg) were weighed and transferred to the SiriusT3 instrument, which automatically added 1.5 mL ISA water and partition solvent (ISA water-saturated octanol). The pH titration (*n* = 3 technical replicates) was performed automatically with acid (0.5 M KCl) and base (0.5 M KOH) solutions.

### 2.6 Calu-3 cell viability

The cytotoxicity of SGC, SGD, MA21 and MA60 in the Calu-3 cell line was evaluated via MTT (3-[4,5-dimethylthiazol-2-yl]-2,5 diphenyl tetrazolium bromide) assay (Berridge and Tan, 1993). Calu-3 cells, a human bronchial cell line, obtained from LGC Standards (Cat No. ATCC-HTB-55) were used from passage number 21–31 for MTT experiments. The cells were seeded in a density of 3 × 10^4^ cells/well in 96 well plates from VWR Chemicals (cat. no. 734–2327, Radnor, USA). After 24 h, the medium was removed and the Calu-3 cells were exposed to SGC (10–80 μg/mL), SGD (40–180 μg/mL), MA21 (200–1600 μg/mL) or MA60 (50–350 μg/mL) for 24 h. This concentration range enabled the determination of CC_50_ values. The pure compounds and the extracts were diluted in medium (10% FBS, 1% P/S) before addition to the cells. Triton-X100 (0.25%) served as a positive control, and cells treated only with medium (10% FBS, 1%P/S) served as a negative control. After 24 h incubation with the test solutions, the supernatants were removed, cell monolayers were washed with 100 μL PBS buffer, and 200 μL of medium and 20 μL of MTT in PBS (5 mg/mL) were applied to each well. After incubation for 3.5 h, the MTT solution was removed and 100 μL DMSO were added to dissolve the formazan crystals. To ensure complete solubilization of the formazan crystals, plates were incubated on a shaking platform (200 rpm) under the exclusion of light for 20 min, and the absorbance was recorded at 570 nm with an Epoch 2 spectrophotometer (BioTek Instruments, Inc., Vermont, USA).

The cell viability was calculated according to Eq. (1).

(1) $$\mathrm{Cell viability}\;\left(\%\right)=\frac{{\mathrm{OD}}_{T}-{\mathrm{OD}}_{P}}{{\mathrm{OD}}_{N}-{\mathrm{OD}}_{P}}\;x\;100$$

where *OD*_*T*_ is the optical density of the test well at 570 nm, *OD*_*P*_ is the optical density of the positive control (no viability) and *OD*_*N*_ is the optical density of the negative control (100% viability).

### 2.7 Permeability

Calu-3 cells were seeded on PET membranes of PS inserts (1.1 cm^2^, 0.4 μm) from Sarstedt (cat. no. 83.3931.040, Nümbrecht, Germany) with a seeding density of 1 × 10^5^ cells/insert, placed in a 12 well plate (cat. no. 734–2324, VWR Chemicals, Radnor, USA). Calu-3 cells were used from passages 36–39 for permeability assays. After approximately two weeks of culturing in a liquid-liquid interface, an intact monolayer with tight junctions formed, which is the requirement for the subsequently performed permeability assays. Monolayer integrity was assessed by two methods: 1) the transepithelial electrical resistance or TEER value and 2) the addition of fluorescein (40 μg/mL). Cell layers with TEER values exceeding 1000 Ω cm^2^ (reached at days 12–14) on the day of the experiment were considered suitable for permeability assays, as was a lack of measurable fluorescein transport. Immediately before permeability studies, the medium was replaced with HBSS and following a 30 min incubation time, TEER values were measured. Permeability studies were performed in a bidirectional manner for SGC, SGD, MA21, and MA60. Metoprolol served as a reference for comparison with available data from the literature. For permeability assessment in the apical to the basolateral direction (A-B), 700 μL of test solutions (SGC/SGD: 20 μg/mL, MA21: 600 μg/mL, MA60: 100 μg/mL in HBSS) were added to the apical compartment and 1700 μL of buffer to the basolateral chamber. For studies from the basolateral to the apical chamber (B-A), 700 μL of buffer was added to the apical compartment and 1700 μL of the same test solutions to the basolateral compartment. 200 μL were withdrawn from donor and acceptor compartments at two time points: directly after the addition of both solutions (*t* = 0 h) and after 120 min (*t* = 2 h). For the duration of the permeability studies, plates were placed on a shaking platform at 37 ± 0.37 °C at 200 rpm. After incubation, cells were carefully washed and the solutions were replaced with HBSS. After a further 30-min incubation, TEER values were again evaluated to assess cell integrity.

The quantitative analysis of permeability samples was performed on a UHPLC-ESI-MS system (Thermo Fisher Scientific, CA) comprising a Dionex UltiMate 3000 coupled to an LTQ XL linear ion trap mass spectrometer, as described in detail in the Supporting information. SGG was used as an internal standard for quantitation (ratio analyte/internal standard). The apparent permeability coefficient (P_app_) was calculated using Eq. (2):

(2) $$P_{\mathrm{app}}=\frac{\mathrm{d}Q}{\mathrm{d}t}+\frac{1}{A*C_{0}}$$

where *dQ/dt* was the amount of analyte present in the acceptor compartment as a function of time, *A* was the surface area of the cell monolayer (1.1 cm^2^) and *C*_*0*_ was the initial concentration of the test compound in the donor compartment. The acceptor compartment in the direction A-B is the basal chamber, and in the direction B-A the apical chamber. P_app_ is calculated in the dimension x 10^−6^ cm/s.

### 2.8 Calu-3 cell uptake

To determine the uptake and retention of SGC and SGD in Calu-3 cells, cells were seeded in DMEM-F12 with 10% FBS at high cell density (5 × 10^5^ cells/cm^2^) in 24-well plates (cat. no. 142475, Thermo Fisher Scientific). The 2D cell monolayer was allowed to equilibrate for 72 h before the fresh medium was supplied, and SGC and SGD were added to culture supernatants at a final concentration of 50 μg/mL. At 4, 12, and 24 h after addition, cell extracts were generated in triplicates. To that end, the medium was aspirated from the respective wells, and cells were washed with pre-warmed ammonium carbonate solution (75 mM, pH 7.4, 37 °C). Immediately after removal of the wash solvent, 300 μL cold extraction solvent (40:40:20 acetonitrile:methanol:water, −20 °C) was added per well to lyse cells in situ and stop the enzymatic activity. The bottom of each well was subsequently scraped and the cell extracts were transferred to 96-well storage plates and stored at −80 °C until analysis. On the day of MS analysis, the cell extract samples were centrifuged at 6 °C for 10 min to remove cell debris, and the cell-free extract was transferred to a fresh 96-well plate for measurement. Quantification of SGC and SGD was performed using flow-injection time-of-flight mass spectrometry (FIA-TOFMS) in negative ion mode as described previously (Fuhrer et al., 2011). SGC and SGD signals were identified by matching accurate masses (for both [M-H]- at 707.2127 *m*/*z*). To account for possible influences of the complex matrix in the cell extract samples, a matrix-matched series of calibration samples were prepared, in which increasing concentrations of SGC were spiked into extracts of cells that had not previously been treated with either SGC or SGD. The calibration samples were injected alongside the cell extract samples and used to establish a calibration function using linear regression. The ion intensities measured were converted into SGC and SGD concentrations and normalized by the cell number in each sample, which was determined at each time point in separate cell cultures using trypan blue staining and cell counting in a Thermo Fisher Countess II Automated cell counter after trypsin-mediated cell detachment. This procedure eventually yielded estimates of SGC and SGD contents in pg/cell.

### 2.9 Statistical analysis

Data analysis was performed with Microsoft Excel version 2404 and GraphPad Prism version 10.2.3 (403). Results are expressed as mean ± standard deviation (SD). To test for normal distribution the skewness of the data set and the Kolmogorov-Smirnov method were used. A two-sided Student's *t*-test was performed for parametric data sets. For non-parametric data the Mann-Whitney *U* test was used instead. For statistical significance *p* ≤ 0.05 was set as the cut off. Quantitative and statistical analysis of the cellular uptake of SGC and SGD was performed in Matlab 2020b with custom analysis scripts using the Matlab functions *regress* and *t-test2*.

## 3 Results and discussion

### 3.1 pH-dependent solubility profile, pK_a_ and logP

In pharmaceutical research early solubility characterisation helps to overcome potential bioavailability difficulties and to choose the best administration route as well as to plan the appropriate formulation strategy (Arnott and Planey, 2012). Thermodynamic solubility is widely regarded as the gold standard for accurately representing true solubility. It defines the maximum amount of a solid compound that can dissolve in a solvent at equilibrium. This method is based on saturating the solvent with excess compound until the dissolution equilibrium is attained. Factors such as buffer composition, time, temperature, mixing conditions, and polymorphism can influence the results (Alsenz and Kansy, 2007; Veseli et al., 2019). The European Medicines Agency (EMA) recommends obtaining biopharmaceutically relevant drug solubility data within a physiologically acceptable pH range (International Council for Harmonisation of Technical Requirements for Pharmaceuticals for Human Use. ICH Harmonised Guideline: Biopharmaceutics Classification System-Based Biowaiver (M9), 2019). pH-dependent solubility profiles refer to solubility at a given pH, measured in a defined pH-buffered system. The solubility of a substance may change in response to variations in pH caused by factors such as the degree of ionisation. This phenomenon can lead to significant differences in the solubility of ionised and non-ionised molecules at a defined pH value (Bergström et al., 2004; Veseli et al., 2019).

**Fig. 2:**
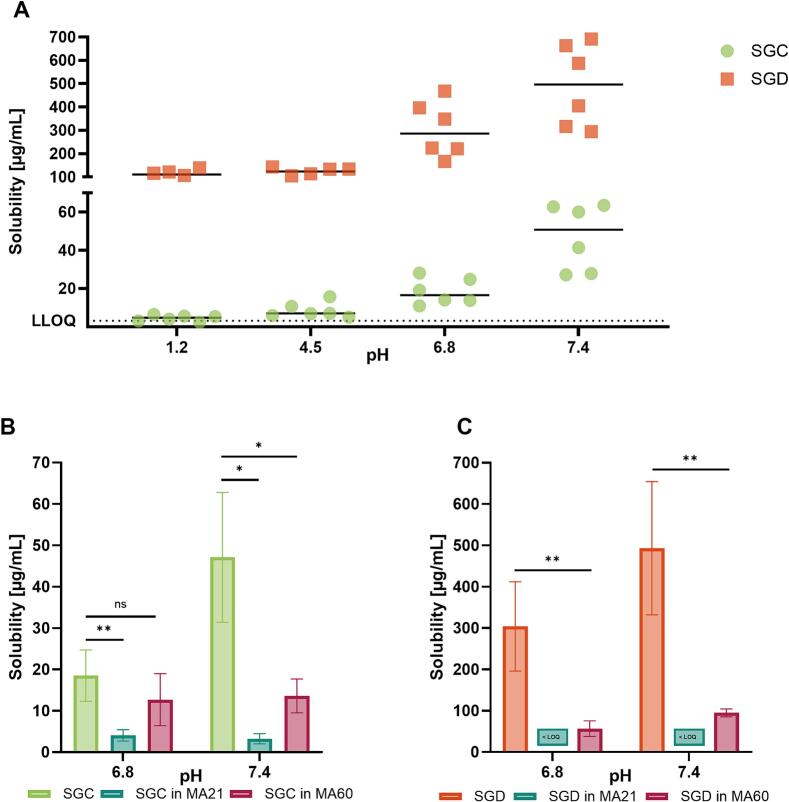
A) Solubility of sanggenon C (SGC, in green) and sanggenon D (SGD, in orange) in four different buffer solutions with pH values of 1.2, 4.5, 6.8 and 7.4 (*n* = 6; black lines indicate the mean values). Solubility of SGC in B) and solubility of SGD in C), both at pH 6.8 and pH 7.4 compared to their solubility in extracts MA21 and MA60 at the same conditions expressed as the mean ± SD (*n* = 3). Values for SGD in MA21 at respective pH values were lower than the LOQ. (For interpretation of the references to colour in this figure legend, the reader is referred to the web version of this article.)

The assessment of pH-dependent solubility of the isolated stereoisomers, SGC and SGD, showed a distinct increase with ascending pH values for both compounds, indicating progressive ionisation of the hydroxyl groups over the range of acidic to neutral pH (Fig. 2A). Furthermore, SGD exhibited an approximately 10-fold higher solubility over SGC at all pH values tested, indicating a profound effect of the steric orientation on the solubility. This was unexpected in light of the high structural similarity between the two stereoisomers. However, differences in solubility for similar flavonoid stereoisomers have been mentioned in the literature (Zheng et al., 2020). Also, the influence on the solubility based on the torsion angle of ring C and B in the flavonoid structure has been described before (Chebil et al., 2007).

The solubility of SGC and SGD within the two multi-component extract mixtures, i.e. MA21 and MA60, at the two neutral pH values, 6.8 and 7.4 (Fig. 2B-C) showed in some cases a significant decrease in solubility compared to the isolated compounds. This is an unusual observation in the field of natural product research, where the opposite behaviour, i.e., a solubility enhancement of APIs due to endogenous solubilization enhancers, is more often reported (Zhao et al., 2020). An increase in water solubility in the presence of a Puerariae radix extract was found for daidzin by Keung and colleagues (Keung et al., 1996). Similarly, the dissolution rate of hypericin was increased by coexisting flavonoids in a *Hypericum perforatum* crude extract (Jürgenliemk and Nahrstedt, 2003). In other examples, an increased solubility of berberine was observed in the presence of Coptidis rhizoma extract (Ma et al., 2016) and the solubility of schizandrin was increased when tested in a complex decoction preparation derived from Schisandrae fructus (Xu et al., 2007).

The lower solubilities of SGC and SGD at acidic pH values might be an explanation for the low serum and tissue concentrations reported by Langeder et al. after oral administration to mice, even at high dose concentrations of up to 100 mg/kg (Langeder et al., 2023b). Even increases in stomach pH caused by the intake of food may not necessarily improve the pH-dependent solubility of SGC and SGD in the stomach following oral administration (Dressman et al., 1990; Langeder et al., 2023b; Simonian et al., 2005). In contrast, the pH of the respiratory tract has been reported to be approximately pH 6.8 (Effros and Chinard, 1969) which provides more favourable dissolution conditions for MDAAs administered via the pulmonary route, at least in terms of pH-effect alone.

To date, no studies have reported experimentally determined values for the ionisation constant (K_a_) of MDAAs, including SGC and SGD. Since the pH-dependent protonation state not only affects physicochemical properties such as solubility, dissolution rate and lipophilicity, but also ADME properties, such as permeability (Manallack et al., 2013), this data can be essential and useful for the prediction of API behaviour in the physiological milieu. There are currently different pK_a_ prediction tools available, such as pKalc, ADMET Predictor, ACD/pK_a_ or Marvin, to gain first insights into the ionisation behaviour of test compounds (Manallack et al., 2013; Prankerd, 2007). When dealing with the comparably large and complex chemical structures of natural product-derived compounds, like SGC and SGD which both have eight hydroxyl groups, in silico pK_a_ prediction can be a challenge because each functional group of these multi-protic molecules has a specific pK_a_ value (Navo and Jiménez-Osés, 2021). In both sanggenons, the respective hydroxyl groups are present at the positions C-3, C-5, and C-7 of the benzopyrone structure as well as at positions C-10″, C-12″, C-16″, and C-18″ connected to the flavonoid core via the tetrasubstituted cyclohexene ring and at position C-4′ in ring B (Fig. 1). Calculated pK_a_ values, generated via MarvinSketch, range from 7.26 to 11.04 (Table 1), with no possibility to differentiate between the two stereoisomers SGC and SGD.

**Table 1:** Experimentally determined (SiriusT3) and calculated (*MarvinSketch, ChemAxon) pK_a_ (*n* = 5) and logP (*n* = 3) values for sanggenon C (SGC) and sanggenon D (SGD) presented as the mean ± S.D.

Property	SGC (experimental)	SGD (experimental)	SGC and SGD (calculated)
pK_a_ 1	2.64 ± 0.69	3.27 ± 0.37	7.26
pK_a_ 2	5.20 ± 0.80	6.37 ± 0.13	7.90
pK_a_ 3	6.74 ± 0.14	7.25 ± 0.32	8.55
pK_a_ 4	7.97 ± 0.06	7.99 ± 0.26	8.96
pK_a_ 5	9.00 ± 0.04	8.99 ± 0.08	9.32
pK_a_ 6	9.84 ± 0.05	9.94 ± 0.04	9.74
pK_a_ 7	10.70 ± 0.02	10.78 ± 0.01	10.46
pK_a_ 8	12.05 ± 0.07	12.04 ± 0.07	11.04
*logP*	3.48 ± 0.05	2.92 ± 0.07	*–*

A total of eight pK_a_ values were measured for SGC and SGD with the UV-metric standard titration method (SiriusT3 apparatus) and compared to in silico determined values (MarvinSketch, ChemAxon; Table 1). Due to the complex structures of SGC and SGD, it was not possible to distinguish and assign the eight pK_a_ values to the eight proton positions. However, it was observed that four out of eight experimentally determined pK_a_ values were substantially lower than the in silico results. Further studies, including a set of derivatives with the removal of the respective phenolic hydroxy groups, would be needed to fully assign the pK_a_ to the respective functional group (Fuguet et al., 2023). However, the mean absolute error (MAE) for the pK_a_ of SGC and SGC are 1.47 and 1.21, respectively, when comparing the pK_a_ values closest to each other. This discrepancy may be caused by the fact that the MarvinSketch algorithm only considers molecular structures and their functional groups when predicting pK_a_ values, without considering the potential influence of adjacent groups. Difficulties in predicting the pK_a_ values of drug-like molecules can be challenging due to tautomerization, heterocycles, conformational flexibility of large molecules, the ability to form intramolecular hydrogen bonds and the presence of various titratable sites since the protonation state of one functional group can affect the proton dissociation tendency of adjacent groups (Işik et al., 2018). The experimental results did roughly correspond with the solubility data where an increased solubility was observed at pH 6.8 and pH 7.4, suggesting that higher deprotonation ratios of both sanggenons at higher pH levels lead to higher solubility. However, differences in pK_a_ values and therefore ionisation states of SGC and SGD cannot be completely responsible for the 10-fold higher solubility of SGD compared to SGC, since the first three pK_a_ values of SGC were significantly lower than the corresponding values of SGD, which would theoretically suggest that under acidic pH conditions, a higher fraction of the SGC molecule should carry anionic charges and therefore be more soluble. Since we observe the opposite, we infer other mechanisms (e.g. tautomerization, conformational state, intramolecular hydrogen bond formation) to be more dominant. These results underscore the importance of experimentally assessing pK_a_ values to elucidate their impact on physico-chemical features, as prediction models still fall short, especially for natural products.

Lipophilicity is also a key factor influencing the pharmacokinetic behaviour of drug substances, including the transfer across cell membranes, distribution kinetics into tissues, absorption, toxicity and protein binding characteristics of a drug, as well as solubility characteristics in aqueous environments (Gleeson, 2008; Hughes et al., 2008; Testa et al., 2000). Thus, the evaluation of lipophilicity is a standard parameter in biopharmaceutical profiling (Arnott and Planey, 2012). Lipophilicity is typically characterized by the logarithmic partition coefficient (logP) between octanol and water (Leo et al., 1971), whereby high logP values (usually >3) represent a higher lipophilicity (Arnott and Planey, 2012). The experimentally determined logP values of SGC and SGD (Table 1) were in the moderately lipophilic range (∼3–3.5), and SGC partitioned to a greater extent in octanol compared to SGD, which also aligns well with the solubility data.

### 3.2 Cytotoxicity and uptake in Calu-3 cells

**Fig. 3:**
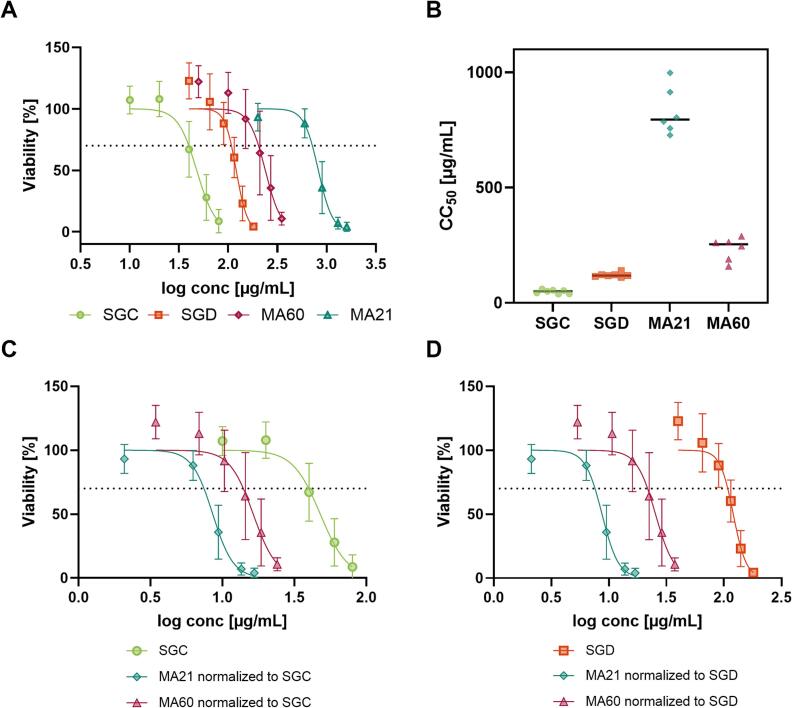
A) Calu-3 cell viability after 24 h in the presence of sanggenon C (SGC), sanggenon D (SGD), MA21 and MA60. Reduction under 70% of viability was considered cytotoxic (dotted line). B) CC_50_ values of SGC, SGD, MA21 and MA60. MA21 and MA60 cell viability data was also plotted against the respective concentrations of (C) SGC and (D) SGD in each extract mixture to assess whether SGC or SGD were the main cytotoxic constituents. Data is expressed as the mean ± SD of *n* = 6 separate experiments with different passage numbers.

Calu-3 cell viability was evaluated via an MTT assay to determine suitable sample concentrations for subsequent permeability investigations. Calu-3 cells, human bronchial epithelial cells, are commonly used for drug transport studies in the lung (Forbes and Ehrhardt, 2005; Mathias et al., 2002). In line with the results of the physicochemical characterisation studies, the stereoisomers SGC and SGD also showed different cytotoxicity profiles, with SGC exhibiting a higher cytotoxicity (Fig. 3A-B). The respective half-maximal cytotoxic concentrations (CC_50_) were calculated from the dose-response curves giving a CC_50_ value of 47.8 μg/mL for SGC and 120.5 μg/mL for SGD (Fig. 3A-B). The observation that SGC is more cytotoxic than SGD is supported by the high logP of SGC indicating that it can permeate more easily across cell membranes compared to SGD.

Exposure of Calu-3 cells to the multi-component extract mixtures, which have a lower content of total MDAAs (MA21 contains 2.6% and MA60 contains 29.0% MDAAs in total) also provided interesting insights. Extract MA60, with a 10-fold higher total sanggenon content, exhibited greater cytotoxicity (CC_50_ = 238.1 μg/mL) compared to the hydroethanolic extract MA21 (CC_50_ = 822.8 μg/mL; Fig. 3A-B), indicating that increased levels of sanggenons correlate with higher cytotoxicity. When MA21 and MA60 cell viability data are plotted against the respective concentrations of SGC and SGD in each extract (Fig. 3B-C) both extracts demonstrated greater cytotoxicity compared to the individual sanggenons. This indicates the likely presence of other unique and currently unidentified compounds in each extract which may contribute to cytotoxicity. Theoretically, if either SGC or SGD were the main cytotoxic component in the mixture, all three curves should overlap. The mismatch of the curves therefore implies that further constituents in the multi-component mixture enhance cytotoxic effects.

**Fig. 4:**
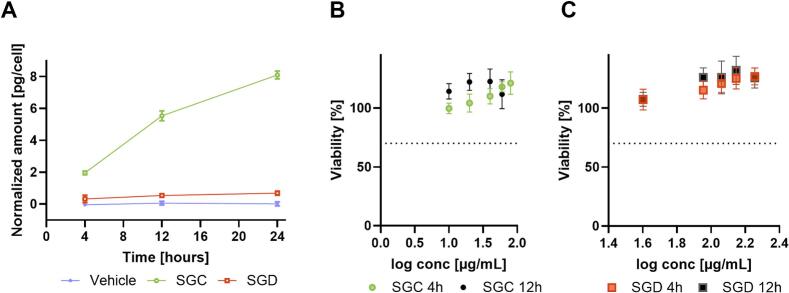
A) Cellular uptake of SGC and SGD in Calu-3 cells. Cell monolayers were incubated with 50 μg/mL compound or vehicle (0.25% DMSO) and cell extracts were generated at 4, 12 and 24 h after washing cell layers with ammonium carbonate solution (75 mM, pH 7.4, 37 °C) to remove residual medium. SGC and SGD contents were additionally normalized to the number of cells, obtained by cell counting. Data points represent the mean ± standard deviation of three replicates. Calu-3 cell viability after 4 h and 12 h treatment with sanggenon C (SGC) (B) and sanggenon D (SGD) (C). Reduction under 70% of viability was considered cytotoxic (dotted line).

To rule out that SGC and/or SGD are retained within the epithelial cells and thereby prevented to exert their full therapeutic properties in the extracellular space (e.g., neuraminidase inhibition, inhibition of pneumococcal biofilm formation), an assessment of sanggenon uptake in Calu-3 cell monolayers was performed. Calu-3 cells seeded at high confluence were exposed to either SGC or SGD after an initial equilibration period of 72 h. For both SGC and SGD, the concentration tested was 50 μg/mL, at which no cytotoxic effects were observed during a 4-h and 12-h incubation period evaluated via MTT assay (Fig. 4B). After 4, 12 and 24 h, the cell layer was washed with ammonium carbonate solution, before cells were extracted by cold solvent extraction for the analysis of intracellular SGC and SGD contents by mass spectrometry. The analysis of the cell extract samples revealed that SGC showed a distinctly higher accumulation than SGD within Calu-3 cells already at 4 h after addition (2.0 ± 0.1 vs. 0.3 ± 0.2 pg/cell, Fig. 4A), which continued to increase until the intracellular SGC content reached a concentration of 8.1 ± 0.3 pg/cell after 24 h (Fig. 4A). In contrast, SGD levels only marginally increased during the same 24 h period indicating that SGC shows greater penetration of Calu-3 cells, consistent with its higher lipophilicity compared to SGD (higher logP, Table 1) facilitating its entry through the cell membrane. Intriguingly, the low intracellular accumulation of SGD prompts the hypothesis that SGD would remain in the extracellular/apical space and neither enters nor permeates the epithelial barrier. Thus, despite their structural similarity, SGC and SGD exhibit different permeation behaviour, and may thus localize differently at the infection site.

### 3.3 Bidirectional permeability of SGC and SGD

Unlike orally administered drugs which generally aim for a high systemic bioavailability and therefore benefit from a high permeability, many inhaled drugs, including anti-infective agents for the treatment of lung infections, have local targets in the lung (Gontijo et al., 2014b; Marchand et al., 2015, Marchand et al., 2016, Marchand et al., 2018). Consequently, a low-to-moderate permeability is desirable for inhaled drugs, since such compounds are permeable enough to reach their targets, but at the same time do not diffuse too quickly from the lung into the plasma, where they are less effective and can cause systemic side effects (Brillault and Tewes, 2020; Guo et al., 2021; Hastedt et al., 2022; Zhou et al., 2015). Fang and colleagues analysed the relationship between flavonoid structure and the ability to permeate through a Caco-2 cell layer and found that passive diffusion is the major transport mechanism for this compound class. Moreover, they described a broad range of apparent permeability coefficients (0.29 ± 0.01 × 10^−6^ cm/s to 33.90 ± 3.55 × 10^−6^ cm/s in direction A-B, and 0.23 ± 0.03 × 10^−6^ cm/s to 42.19 ± 3.11 × 10^−6^ cm/s in direction B-A) for different tested flavonoids such as myricetin, daidzein, and kaempferide (Fang et al., 2017). In many cases, a low permeability of flavonoids has been reported (Zhao et al., 2019). Interestingly, PK synergies between coexisting constituents of natural product extracts have been reported to increase or decrease the permeability of plant constituents (Machado et al., 2020). For example, improved permeability behaviour was found for the flavonoid baicalin when present as constituent of the root extract of *Angelica dahurica* (Zhu et al., 2013). In contrast, a decreased permeability has been reported for alkaloids from Ephedrae herba in different plant extracts on the transport compared to pure alkaloid compounds (Zheng et al., 2015). In literature, there are also examples described with no changes in permeability due to matrix effects; e.g., no influence of the *Prunella vulgaris* extract matrix was observed for the permeability of rosmarinic acid in Caco-2 cells. Also, ursolic acid, a pentycyclic triterpene, showed no difference in permeability behaviour in the same cell line, when comparing the results obtained from the isolated compound and as constituent in an extract of *Salvia officinalis* (Qiang et al., 2011). The different examples demonstrate that it is not possible to extrapolate PK synergy in natural products, thus making experimental investigations indispensable.

**Fig. 5:**
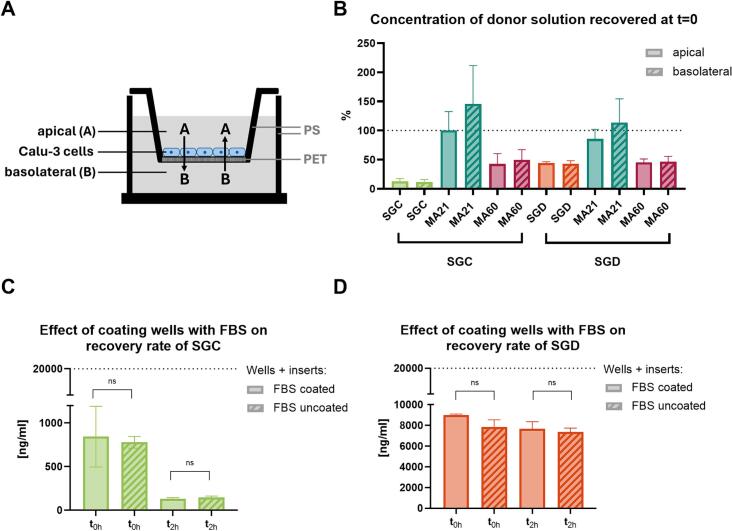
A) Shows the permeability setup. Calu-3 cells are cultured on a membrane, thereby creating a barrier between the acceptor and donor chamber (well plate insert: membrane material: polyethylene therephthalate (PET), frame material: polystyrene (PS) and well plates: PS). B) Recovered SGC and SGD concentrations in the donor compartment in the t = 0 h control samples. Control experiment for serum-binding evaluated for sanggenon C (SGC; C) and sanggenon D (SGD; D). The dotted line represents the applied concentration. Values represent the mean ± standard deviation of *n* = 3 technical replicates.

The permeability evaluation of SGC, SGD, MA21, and MA60 was conducted bidirectionally across a Calu-3 monolayer. For this setup (Fig. 5A), the test sample was added to either the apical chamber (in the A-B direction) or the basolateral chamber (in the B-A direction) at subtoxic concentrations: SGC (20 μg/mL), SGD (20 μg/mL), MA21 (600 μg/mL), and MA60 (100 μg/mL). The concentration of permeated SGC or SGD was subsequently determined in the acceptor compartment after a 2-h incubation period. As a routine control, a small volume of donor solution was taken immediately after addition to the donor compartment (*t* = 0 h). We observed an unexpected reduction in the SGC and SGD concentrations in the donor compartment in our t = 0 h control samples (Fig. 5B). The reduction in concentration was more pronounced for SGC than SGD, possibly indicating the binding of the more hydrophobic compound either to residual proteins in the well from the serum-supplemented medium or to the plastic components of the wells themselves. Control experiments showed no indication that SGC or SGD bind to serum proteins. However, both SGC and SGD might interact with certain plastic materials (Fig. 5C-D). As neither the molecular structures nor the logP values of the sanggenons were indicative of a high adsorptive behaviour of these compounds to different surfaces, this observation is important, especially for the design of future studies with this class of compounds.

Despite the significant reduction of the free compounds in the donor chamber at t = 0 h, the remaining concentration of free drug was sufficiently high to maintain sink conditions and be detectable in small amounts above the limit of quantitation. Metoprolol (500 μM) served as a reference compound to distinguish high from low permeability and ensure consistency of results with the literature (International Council for Harmonisation of Technical Requirements for Pharmaceuticals for Human Use. ICH Harmonised Guideline: Biopharmaceutics Classification System-Based Biowaiver (M9), 2019; Shah and Amidon, 2014; Zur et al., 2014). Bosquillon et al. determined the P_app_ for metoprolol (10 μM in HBSS) in Calu-3 cells with 10.3 ± 0.0 × 10^−6^ cm/s, in 16HBE14o- cells with 24.5 ± 2.5 × 10^−6^ cm/s and in NHBE cells with 11.3 ± 0.7 × 10^−6^ cm/s (Bosquillon et al., 2017). The mean P_app_ values determined for metoprolol in this study were 25.4 ± 6.9 × 10^−6^ cm/s (A-B) and 23.3 ± 4.9 × 10^−6^ cm/s (B-A), indicating a good correspondence with the literature. The slightly higher P_app_ values measured in our study are likely due to higher initial metoprolol concentrations used compared to studies from the literature.

**Fig. 6:**
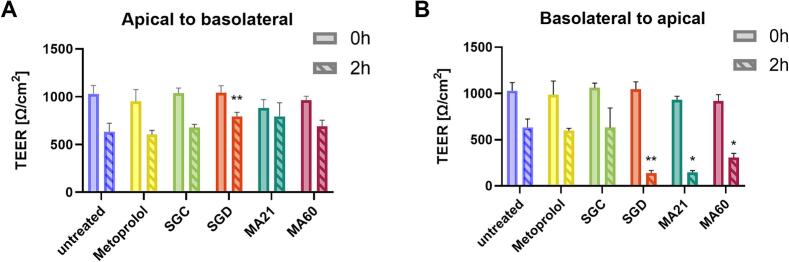
Changes in TEER before and after permeability study in apical to the basolateral direction (A) and basolateral to the apical direction (B) expressed as the mean ± S.D. (n = 3). The statistical analysis was performed using Mann-Whitney *U* test. Comparisons were made between the untreated groups (2 h) and the treated groups (2 h).

Interestingly, SGC and SGD could not be detected in the acceptor chamber after 2 h of incubation (A-B direction). Neither by applying the isolated compounds nor the extracts MA60 and MA21, any evidence for the permeability of SGC or SGD was detectable. Small amounts of SGD (<0.6%) were detected in the acceptor chamber when the B-A permeability was investigated after applying either (i) isolated SGD = 35.4 ± 15.1 ng/mL, (ii) MA21 = 7.9 ± 4.2 ng/mL, or (iii) MA60 = 10.6 ± 5.7 ng/mL (n = 3 experiments with different passage numbers). However, this minor increase in B-A permeability also coincided with a loss in TEER only observed when SGD was added to the basolateral chamber (Fig. 6A-B). The observed decrease in TEER during experiments may result from a loss of epithelial integrity. Cellular damage can be ruled out, as a subsequent MTT assay revealed no cytotoxic effects. Additionally, TEER measurements taken after 24 h of incubating with medium at 37 °C, showed recovery to initial values, suggesting a temporary integrity loss. Control experiments demonstrated that the TEER decrease was specific to this particular batch of Calu-3 cells as it could not be repeated with an independent batch of Calu-3 cells (Fig. S1, Supporting Information). Despite the TEER decrease, permeation of the non-permeation marker, fluorescein, did not increase in these monolayers (Supporting Information). A decrease in TEER exclusively in the B–A direction is rare but has been reported in Calu-3 cells as a property of the mycotoxin, deoxynivalenol (Diesing et al., 2011; Manford et al., 2005) and in 16HBE14o- human bronchial epithelial cell line possibly due to the influence of DMSO (Manford et al., 2005).

### 3.4 Implications of this study for pulmonary delivery of sanggenons and beyond

The relevance of this interdisciplinary study lies in the recognition that biopharmaceutical profiling and in-depth in vitro investigations of PK synergy can provide important insights into (i) whether the purified bioactive compound or the parent extract will perform more favourably in vivo and (ii) which administration route is likely to provide optimal therapeutic activity. This information will help to inform the planning of in vivo proof-of-concept efficacy studies and formulation strategies. In the case of the two isolated MDAA stereoisomers studied here, SGC and SGD, the rare observation was made that the isolated bioactive compounds exhibited more favourable properties compared to the parent extracts (MA21 and MA60), for instance in terms of improved solubility. It is important to note that despite the high structural similarity of the herein investigated stereoisomers, SGD exhibited a 10-fold higher solubility in aqueous medium than SGC at all four tested pH values. This difference in solubility behaviour between stereoisomers is currently not easily predicted by in silico models and thus emphasises the need for experimental studies, such as those described here.

Therapeutic strategies for SGC and SGD targeting the respiratory tract may hold high potential for both preventive and acute antiviral and antibacterial treatments and associated superinfections. This could include antibacterial effects due to the inhibition of biofilm formation, circumvention of virus release from the cells after viral infection and prevention of secondary infections due to neuraminidase inhibition (Grienke et al., 2016; Wasilewicz et al., 2023). Based on the low uptake and permeability in Calu-3 cells, the tested MDAAs are expected to be able to exert their inhibitory effect on neuraminidases of *Streptococcus pneumoniae* and influenza strains, and potentially also in a dual manner, in the extracellular space (Grienke et al., 2016). Also, the inhibition of planktonic growth and biofilm formation of *S. pneumoniae* is estimated as possible due to low permeability and uptake. On the other hand, the previously determined anti-SARS-CoV-2 activities of SGC and SGD (IC_50_ values of 7.2 μM and 13.9 μM, respectively, (Wasilewicz et al., 2023)), which have been reported to be based on inhibition of the viral main protease, could be constrained since the enzyme is localized within the cell (Fan et al., 2004; Wasilewicz et al., 2023).

The results of this study are currently being utilised to plan future in vivo proof-of-concept studies in a murine infection model using intrapulmonary delivery of SGC and SGD formulations. Since the efficacy of anti-infective agents is a complex interplay between PK behaviour at the infection site and efficacy parameters such as the minimal inhibitory concentration (MIC) or minimum biofilm inhibitory concentration (MBIC) it is hypothesized that the possibility of high lung retention of sanggenons, due to their low permeability properties, will be highly favourable for therapeutic efficacy. To ensure the application of the highest possible concentrations, optimized formulation of SGC and SGD will be necessary. Future formulation strategies for inhaled sanggenons should focus on both dry powders and liquid formulations for nebulization.

## 4 Conclusion

Our study showed that biopharmaceutical profiling is a highly useful, albeit currently under-utilised strategy to evaluate the suitability of isolated natural compounds and multi-component mixtures for their intended therapeutic applications. In this study, we were able to show that the two MDAAs, SGC and SGD, demonstrate more favourable PK properties, including e.g. higher solubility as isolated compounds rather than as constituents of multi-component extracts. Furthermore, the low permeability behaviour of SGC and SGD suggests a high potential for lung delivery via inhalation administration to treat acute respiratory tract infections. Ongoing research, including planned in vivo proof-of-concept studies using inhalation administration will show whether the PK advantage of lung delivery for poorly permeable anti-infectives proves therapeutically relevant.

## CRediT authorship contribution statement

**Jacqueline Schwarzinger:** Investigation, Methodology, Visualization, Writing – original draft. **Sigrid Adelsberger:** Investigation, Methodology, Visualization, Writing – original draft. **Karin Ortmayr:** Investigation, Methodology, Visualization. **Sarah Luise Stellnberger:** Investigation, Methodology, Writing – review & editing. **Ammar Tahir:** Methodology. **Gabriela Hädrich:** Investigation, Methodology, Supervision, Writing – review & editing. **Verena Pichler:** Methodology, Supervision, Writing – review & editing. **Judith M. Rollinger:** Funding acquisition, Supervision, Writing – review & editing. **Ulrike Grienke:** Conceptualization, Funding acquisition, Methodology, Supervision, Writing – review & editing. **Lea Ann Dailey:** Conceptualization, Funding acquisition, Supervision, Writing – review & editing.

## Declaration of competing interest

The authors declare no competing financial interest.

## Data Availability

The original contributions presented in the study are included in the article/Supplementary Material, further inquiries can be directed to the corresponding authors.

## Appendix A Supplementary data

Supporting Information Image 1
