# Supplementary material for: Biopharmaceutical profiling of anti-infective sanggenons from Morus alba root bark for inhalation administration
Source: Int J Pharm X. 2024 Aug 5;8:100272. doi: 10.1016/j.ijpx.2024.100272 (PMC11381475; doi:10.1016/j.ijpx.2024.100272)
Supplement: Supplementary file 1 — Supporting Information [file mmc1.docx]

Supporting information

**Biopharmaceutical profiling of anti-infective sanggenons from *Morus alba* root bark for inhalation administration**

*Authors:*

*Jacqueline Schwarzinger^a,b,#^, Sigrid Adelsberger^b,c,#^, Karin Ortmayr^c^, Sarah Stellnberger^b,d^, Ammar Tahir^c^, Gabriela Hädrich^a^, Verena Pichler^d^, Judith M. Rollinger^c^, Ulrike Grienke^c,*^, Lea Ann Dailey^a,*^*

^#^ J. Schwarzinger and S. Adelsberger contributed equally to this work

*Correspondence:*

^*^ U. Grienke ([ulrike.grienke@univie.ac.at](mailto:ulrike.grienke@univie.ac.at)) and L.A. Dailey ([leaann.dailey@univie.ac.at](mailto:leaann.dailey@univie.ac.at))

*Affiliations:*

*^a^ Division of Pharmaceutical Technology and Biopharmaceutics, Department of Pharmaceutical Sciences, Faculty of Life Sciences, University of Vienna, Josef-Holaubek-Platz 2, 1090 Vienna, Austria*

*^b^ Vienna Doctoral School of Pharmaceutical, Nutritional and Sport Sciences, University of Vienna, Josef-Holaubek-Platz 2, 1090 Vienna, Austria*

*^c^ Division of Pharmacognosy, Department of Pharmaceutical Sciences, Faculty of Life Sciences, University of Vienna, Josef-Holaubek-Platz 2, 1090 Vienna, Austria*

*^d^ Division of Pharmaceutical Chemistry, Department of Pharmaceutical Sciences, Faculty of Life Sciences, University of Vienna, Josef-Holaubek-Platz 2, 1090 Vienna, Austria*

**Table of contents**

1. **Sample preparation for quantitation of MDAAs**……………………………………………………4

1.1. pH-dependent solubility profile……………………………………………………………………….4

1.1.1. Preparation of stock and working solutions, calibration standards and QC working samples……………………………………………………….………………………………….4

**Table S1.** Overview of the preparation of stock and working solutions, calibration standards and QC working samples for the pH-dependent solubility assay………….…5

1.1.2. Prearrangement of QC samples………………………...……………..…………………….6

**Table S2**. Prearrangement of QC samples……………………………………………………..….6

1.1.3. Preparation of solubility samples………………….…...………….……………………….6

**Table S3**. Preparation of solubility samples and blanks….………………………………..….6

1.1.4. MTBE-based extraction protocol and completion of sample preparation……….7

**Table S4.** Sequence of the MTBE-based extraction protocol…………………………………7

**Table S5.** Completion of sample preparation before the measurement.…………………7

1.1.5. Evaluation of the MTBE-based extraction protocol……………………………………..8

**Figure S1.** Recovery results for the evaluation of the efficiency of the MTBE-based extraction protocol……………………………………………………………………………………….8

1.1.6. Measurement of solubility samples via UPLC-PDA method………..……………....9

**Table S6.** List of parameters for UPLC-PDA measurement of pH-dependent solubility assay samples……………………………………………………………………………….………….…9

1.1.7. Evaluation of UPLC-PDA quantitation method…………………………..…………....10

**Table S7.** Validation parameters of the UPLC-PDA quantitation method of the pH-dependent solubility assay……………………………………………………………….……….….10

1.2. Bidirectional permeability assay in Calu-3 cells………………………………………………..11

1.2.1. Preparation of stock and working solutions and calibration standards…………11

**Table S8.** Overview of stock and working solutions for the bidirectional permeability assay in Calu-3 cells……………………………………………………………………..………….…11

**Table S9**. Low and high concentration range calibration standard series of the bidirectional permeability assay in Calu-3 cells………………………………………..………12

1.2.2. Preparation of permeability samples………………….…...…………………………….13

**Table S10**. Preparation protocol for low concentration range permeability samples.13

1.2.3. Measurement of permeability samples via UHPLC-ESI-MS method…………….14

**Table S11.** Parameters for UHPLC-ESI-MS measurement of permeability samples………………………………………………………………………………………………….…14

1.2.4. Measurement of metoprolol containing permeability samples via UHPLC-ESI-MS method……………………………………………………………………………………….……….15

**Table S12.** List of method adaptions for metoprolol containing permeability samples compared to sanggenon containing permeability samples…………………………………15

1.2.5. Validation of UHPLC-ESI-MS method…..………………………………………….…....16

**Table S13.** Validation parameters of the UHPLC-ESI-MS quantitation method of the bidirectional permeability in Calu-3 cell assay samples.…………………….………….….16

1.3. Results of the pH-dependent solubility profile………………………………………………….17

**Table S14.** Solubility of sanggenon C (SGC) and sanggenon D (SGD)……………………17

1. **Permeability control experiments**………………………………………………….…………………18
   1. Materials and methods……………………………………………..………………………………..18
   2. Results and discussion……………………………………………………………………………....19 **Figure S2.** Decline in TEER, Papp of fluorescein and cell viability after permeability assay……………………………………………………………………………………………………….21
2. **Results of uptake in Calu-3 cells**………………………………………………….…………………..23

**Table S15.** Results of uptake in Calu-3 cells…………………………………………………….23

1. **Sample preparation for quantitation of MDAAs**

In the following chapters, preparation steps for the quantitation of samples from (a) pH-dependent solubility profile assays and (b) bidirectional permeability in Calu-3 cells testing series are listed. For method validation of (a), quality control (QC) samples have been used, which represent samples with known concentrations of sanggenon C (SGC) and sanggenon D (SGD) that were processed in the same way as test samples. For the evaluation of (b), sanggenon G (SGG) was used as internal standard (IS) to minimize errors such as changes in detector response and to assess the quality of the measurement process.

- 1. **pH-dependent solubility profile**

Different protocols for sample preparation of the pH-dependent solubility profile assays are listed, beginning with descriptions for the production of stock and working solutions up to method evaluation.

- - 1. Preparation of stock and working solutions, calibration standards and QC working samples

Table S1 shows stock and working solutions for calibration curve and QC working samples. Twelve QC working samples containing both SGC and SGD were prepared for low (6.25 µg/mL), middle (25 µg/mL) and high (100 µg/mL) concentration range. For QC blanks, amounts of respective buffer were used. All samples were prepared in the solvent methanol (MeOH).

**Table S1.** Overview of the preparation of stock and working solutions, calibration standards and QC working samples for the pH-dependent solubility assay.

|  | **sanggenon C (SGC)**  **[µg/mL]** | **sanggenon D (SGD)**  **[µg/mL]** |
| --- | --- | --- |
| **calibration curve standards:** | | |
| 2 stock solutions | 1000 | - |
|  | - | 1000 |
| 1 working solution | 400 | 400 |
| 9 standards | 0.78 | 0.78 |
|  | 1.56 | 1.56 |
|  | 3.13 | 3.13 |
|  | 6.25 | 6.25 |
|  | 12.5 | 12.5 |
|  | 25.0 | 25.0 |
|  | 50.0 | 50.0 |
|  | 100 | 100 |
|  | 200 | 200 |
| **QC samples:** | | |
| 2 stock solutions | 1000 | - |
|  | - | 1000 |
| 1 working solution | 400 | 400 |
| 12 working samples | 6.25 | 6.25 |
|  | 25.0 | 25.0 |
|  | 100 | 100 |
|  | 6.25 | 6.25 |
|  | 25.0 | 25.0 |
|  | 100 | 100 |
|  | 6.25 | 6.25 |
|  | 25.0 | 25.0 |
|  | 100 | 100 |
|  | 6.25 | 6.25 |
|  | 25.0 | 25.0 |
|  | 100 | 100 |

- - 1. Prearrangement of QC samples

For the prearrangement of QC samples, 80 µL of the respective QC working samples were used, following the protocol described in Table S2.

**Table S2.** Prearrangement of QC samples

| - 80 µL of the QC working sample were pipetted into a 1.5 mL Eppendorf tube |
| --- |
| - Evaporation of MeOH via GeneVac |
| - 80 µL of respective buffer solution were added |
| - Shaking on a vortex shaker for 60 min |
| - Ultrasonication for 10 min including cooling of the bath with ice pads |
| - Centrifugation in a mini centrifuge for 30 s with 5400 rpm |
| - Neutralization with 0.1% aqueous ammonia solution depending on the respective buffer - 90 µL for buffer pH 1.2 - 80 µL for buffer pH 4.5 - 30 µL for buffer pH 6.8 - sample with buffer pH 7.4 were not manipulated |
| - Vortexing for 10 s |
| - Centrifugation for 30 s with 5400 rpm |

- - 1. Preparation of solubility samples

Table S3 describes the preparation protocol for solubility samples (volume 80 µL). For setting up of blanks, 80 µL of respective buffer solution in a 1.5 mL Eppendorf tube were processed in the same way, beginning with neutralization. After these steps, the application of the MTBE (methyl tertiary butyl ether)-based extraction protocol was possible.

**Table S3.** Preparation of solubility samples and blanks.

| - Centrifugation for 15 s with 5400 rpm |
| --- |
| - Neutralization with 0.1% aqueous ammonia solution depending on the respective buffer - 90 µL for buffer pH 1.2 - 80 µL for buffer pH 4.5 - 30 µL for buffer pH 6.8 - sample with buffer pH 7.4 were not manipulated |
| - Vortexing for 10 s |
| - Evaporation of MeOH |

- - 1. MTBE-based extraction protocol and completion of sample preparation

The MTBE-based extraction protocol was implemented as a three-cycle procedure with 3 x 750 µL as extraction solvent and can be seen in Table S4. This procedure was followed for all samples, blanks, QC samples and QC blanks.

**Table S4.** Sequence of the MTBE-based extraction protocol.

| - Addition of 750 µL MTBE to the 1.5 mL Eppendorf tube containing the sample |
| --- |
| - Shaking on a vortex shaker for 60 min |
| - Ultrasonication for 10 min including cooling of the bath with ice pads |
| - Centrifugation for 30 s with 5400 rpm |
| - Removal (after pipetting up and down 5 times) and transfer of 650 µL supernatant into a second 1.5 mL Eppendorf collector tube |
| - Removal of the solvent in both Eppendorf tubes via sample concentrator |
| - Start of next cycle with addition of 750 µL MTBE or storage of the sample at -20 °C after cycle 3 |

Shortly before the measurement, the completion of sample preparation was done in accordance with the protocol in Table S5.

**Table S5.** Completion of sample preparation before the measurement.

| - Thawing of the sample at room temperature |
| --- |
| - Addition of 80 µL MeOH |
| - Vortexing for 10 s |
| - Shaking for 10 min |
| - Ultrasonication for 10 min |
| - Centrifugation for 30 s with 5400 rpm |
| - Pipetting of the sample into a 300 µL HPLC vial insert |

- - 1. Evaluation of the MTBE-based extraction protocol

The MTBE-based extraction protocol was the basis for the preparation of all samples of the pH-dependent solubility assay. Therefore, the method was validated, including 12 different QC combinations of pH values (1.2, 4.5, 6.8, 7.4) and concentrations (6.25, 25 and 100 µg/mL), available in 3, 4 or 6 replicates. For pH 1.2 in low concentration range only duplicates were definable and drawn upon calculation.

To show the extraction efficiency for this extraction protocol, recovery was calculated as percentage of the known analyte amount for both SGC and SGD. Figure S1 shows the recovery results for both SGC and SGD.

**Figure S1.** Recovery results for the evaluation of the efficiency of the MTBE-based extraction protocol as part of the pH-dependent solubility assay. Left for sanggenon C (SGC), right for sanggenon D (SGD).

Over all parameters, the results for SGC were higher than for SGD, except for 100 µg/mL at pH 7.4. For compound SGC, the recovery value means were in the acceptable range of ± 15%, with exception of 25 µg/mL at pH 7.4 (18% deviation). For compound SGD the data availability was more complex with good recovery of ± 15% for pH 1.2 and pH 6.8. However, recovery results for pH 4.5 and pH 7.4 for SGD were partly too low. The lowest result was found for pH 4.5 and 6.25 µg/mL (-28% from expected value).

- - 1. Measurement of solubility samples via UPLC-PDA method

Quantitative analysis measurements were performed on a Waters Acquity UPLC H-class system using a PDA detector at 205 nm wavelength. Parameters of the measurement are listed in Table S6.

**Table S6.** List of parameters for UPLC-PDA measurement of pH-dependent solubility assay samples.

| device | Waters Acquity UPLC H-Class system |
| --- | --- |
| detector | PDA, wavelength 205 nm |
| autosampler temperature [°C] | 8 |
| injection volume [µL] | 5 |
| mobile phase | double-distilled water with 0.1% FA (solvent A)  acetonitrile with 0.1% FA (solvent B) |
| column | Acquity BEH Phenyl column  2.1 x 100 mm, 1.7 µm |
| column temperature [°C] | 40 |
| flow rate [mL/min] | 0.3 |
| gradient program [time (min)/% B] | 0/5, 1/50, 6.9/50, 7/98, 8.9/98, 9/5, 10/5 |
| total run time [min] | 10 min |
| equilibration time before next injection [min] | 2.0 min |
| data acquisition and processing | Waters Empower 3 |

- - 1. Evaluation of UPLC-PDA quantitation method

Linearity for solubility quantitation method was calculated via regression equation for nine calibration levels (0.78, 1.56, 3.13, 6.25, 12.5, 25, 50, 100 and 200 µg/mL in methanol) for both SGC and SGD. A visually evaluated signal-to-noise ratio higher than 3 times allowed the evaluation of limit of detection (LOD), higher than 10 times the limit of quantitation (LOQ). Intra-day precision was calculated via QC samples, if possible, with n = 6 replicates. The inter-day precision was calculated via repeated measurement of QC samples on three different days. The number of available replicates for the solubility samples ranged from 3–6. Table S7 gives an overview of obtained validation parameters.

**Table S7.** Validation parameters of the UPLC-PDA quantitation method of the pH-dependent solubility assay.

|  | **sanggenon C (SCG)** | **sanggenon D (SGD)** |
| --- | --- | --- |
| levels used | 9 | 9 |
| linear range [µg/mL] | 0.78–200 | 0.78–200 |
| R^2^ | 0.9998 | 0.9983 |
| LOD [µg/mL] | 1.56 | 1.56 |
| LOQ [µg/mL] | 3.13 | 3.13 |
|  | | |
| Precision as standard deviation | | |
| intra-day measurement [%] | 0.4–4.1 | 0.7–3.6 |
| intra-day QC samples [%] | 0.1–21.3 | 0.0–17.1 |
| inter-day QC samples [%] | 0.8–25.0 | 2.8–25.0 |

- 1. **Bidirectional permeability assay in Calu-3 cells**

This chapter describes different protocols for sample preparation of the bidirectional permeability assay in Calu-3 cells including an overview of stock and working solutions, sample preparation protocols, measurement parameters and method validation.

- 1. gg
     1. Preparation of stock and working solutions and calibration standards

For the quantitation of samples from the bidirectional permeability assay in Calu-3 cells, the preparation of stock solutions containing SGC, SGD and SGG, all in methanol, was necessary. The first two stock solutions have been used for calibration curve dilution series, the latter was used for internal standard (IS) purposes. Table S8 allows an overview of stock and working solutions.

**Table S8.** Overview of stock and working solutions for the bidirectional permeability assay in Calu-3 cells.

|  | **sanggenon C (SGC)**  **[µg/mL]** | **sanggenon D (SGD)**  **[µg/mL]** | **sanggenon G (SGG)**  **[µg/mL]** |
| --- | --- | --- | --- |
| **calibration curve standards:** | | |  |
| 2 stock solutions | 1000 | - | - |
|  | - | 1000 | - |
| 2 working solutions | 100 | 100 | - |
|  | 10 | 10 | - |
| **internal standard (IS):** | | | |
| 1 stock solution | - | - | 1000 |
| 3 working solutions | - | - | 100 |
|  | - | - | 10 |
|  | - | - | 0.5 |

Based on knowledge from pre-trials, concentration range of SGC and SGD in permeability samples was expected to be extensive, from low ng range to high µg range. Therefore, standards for two different calibration curve rows (high concentration, low concentration) were prepared. Both series were spiked with IS (100 ng/mL for low concentration range and 1000 ng/mL for high concentration range).

**Table S9.** Low and high concentration range calibration standard series of the bidirectional permeability assay in Calu-3 cells.

|  | **sanggenon C (SGC)**  **[ng/mL]** | **sanggenon D (SGD)**  **[ng/mL]** | **sanggenon G (SGG)**  **[ng/mL]** |
| --- | --- | --- | --- |
| **Low concentration range calibration standards** | | |  |
| 8 levels for SGC, 6 levels for SGD | 3.13 | 3.13 | 100 |
|  | 6.25 | 6.25 | 100 |
|  | 12.5 | 12.5 | 100 |
|  | 25.0 | 25.0 | 100 |
|  | 50.0 | 50.0 | 100 |
|  | 75.0 | 75.0 | 100 |
|  | 150 | - | 100 |
|  | 200 | - | 100 |
| **High concentration range calibration standards** | | | |
| 8 levels for SGC, 7 levels for SGD | 19.5 | 19.5 | 1000 |
|  | 39.1 | 39.1 | 1000 |
|  | 78.1 | 78.1 | 1000 |
|  | 156.3 | 156.3 | 1000 |
|  | 312.5 | 312.5 | 1000 |
|  | 625 | 625 | 1000 |
|  | 1250 | 1250 | 1000 |
|  | 2500 | - | 1000 |

- - 1. Preparation of permeability samples

For the high concentration range, 10 µL of the homogenized permeability samples were spiked with 10 µL of 10 µg/mL IS in methanol and filled up with 80 µL methanol. Thus, dilution factor for each high range sample was 1:10. Blanks for this concentration range were prepared by use of 10 µL pure HBSS.

Table S10 describes the sample preparation for low concentration range samples and respective blanks. Hence, 200 µL of the sample have been dissolved in 50 µL solvent only, the concentration factor for each sample in the low concentration range was 4:1. To ensure the same matrix conditions as in the permeability samples, the low concentration range calibration curve standards were provided in vials containing residue of 200 µL evaporated HBSS buffer.

**Table S10.** Preparation protocol for low concentration range permeability samples.

| - Addition of 10 µL of 0.5 µg/mL IS in methanol to 200 µL of the sample (or to 200 µL pure HBSS for blanks) available in a 1.5 mL Eppendorf tube |
| --- |
| - Vortexing for 10 s |
| - Evaporation of the solvent via GeneVac device |
| - Addition of 50 µL of a methanol:double distilled water = 1:1 mixture |
| - Vortexing for 10 s |
| - Shaking for 15 min |
| - Centrifugation for 2 s with short spin function of a mini centrifuge |
| - Transfer of the sample into a 300 µL HPLC vial insert |

- - 1. Measurement of permeability samples via UHPLC-ESI-MS method

Quantitative analysis measurements were performed on a UHPLC-ESI-MS system (Thermo Fisher Scientific, CA) containing a Dionex UltiMate 3000 coupled to a LTQ XL linear ion trap mass spectrometer. Calibration curves for sanggenon C (SGC) included 8 levels for the low concentration range and 8 levels for the high concentration range; calibration curves for sanggenon D (SGD) were calculated based on 6 levels for the low and 7 levels for the high concentration range (see Table S9). Sanggenon G (SGG) was used as an internal standard for quantitation (ratio analyte/internal standard). Parameters of the measurement can be found in Table S11. The total run time for each sample was 7 min, with a gradient step of 10% solvent A for 1.0 min at the beginning to get rid of buffer constituents, followed by 6 min of actual separation. To avoid buffer constituents transport to the MS detector, the divert valve setting of the LTQ XL linear ion trap device included a transfer of the flow to the waste container in the first 2.41 min and from 5.06 min to 6.52 min. To enhance the sensitivity of the quantitative analysis, single ion monitoring recording was chosen as scanning mode for the MS detector for [M-H]- with two narrow m/z windows: 692.20–694.20 for internal standard SGG and 706.23–708.23 for compounds SGC and SGD.

**Table S11.** Parameters for UHPLC-ESI-MS measurement of permeability samples.

| chromatographic separation and detection | Dionex UltiMate 3000 coupled to a  LTQ XL linear ion trap mass spectrometer |
| --- | --- |
| autosampler temperature [°C] | 12 |
| injection volume [µL] | 5 |
| mobile phase | double-distilled water with 0.1% FA (solvent A)  acetonitrile with 0.1% FA (solvent B) |
| flow rate [mL/min] | 0.35 |
| gradient program [time (min)/% B] | 0/10, 0.99/10, 1/50, 3/75, 3.5/98, 4.5/98, 4.6/50, 7/50 |
| column | Acquity BEH C_18_ column, 2.1 x 50 mm, 1.7 µm |
| column temperature [°C] | 50 |
| total run time [min] | 7 min |
| equilibration time before next injection [min] | 1.6 min |
| ionisation | negative mode |
| HESI source heater temperature [°C] | 400 |
| sheath gas/aux gas/sweep gas flow rates [arbitrary units] | 35/20/0.02 |
| source voltage [kV] | 2.5 |
| capillary temperature [°C] | 400 |
| data acquisition and processing | XCalibur software (version 4.27.0.19) |

- - 1. Measurement of metoprolol containing permeability samples via UHPLC-ESI-MS method

Due to different molecular weight of metoprolol compared with sanggenons, metoprolol containing permeability samples were analyzed in a separate UHPLC-ESI-MS measurement. The same device combination and parameters for permeability samples were used as described in chapter 1.2.3, but with some adaptions that are listed in Table S12.

**Table S12.** List of method adaptions for metoprolol containing permeability samples compared to sanggenon containing permeability samples.

| gradient program [time (min)/% B] | 0/10, 1.5/98, 2.3/98, 2.4/10, 4.5/10 |
| --- | --- |
| column | Kinetex C_18_ column, 150 x 2.1 mm, 1.7 µm, 100 Å |
| Injection volume [µL] | 0.1 |
| total run time [min] | 4.5 min |
| ionisation | positive mode |
| sheath gas/aux gas/sweep gas flow rates [arbitrary units] | 35/15/2 |
| source voltage [kV] | 3.5 |
| capillary temperature [°C] | 400 |
| data acquisition and processing | XCalibur software (version 4.27.0.19) |

Single ion monitoring recording was set to m/z values 266.30–270.20 for [M+H]+. To avoid buffer constituent transport to the MS detector, the divert valve setting was changed to source inlet status from only 2.80 min to 3.50 min.

- - 1. Validation of UHPLC-ESI-MS method

Validation parameters of the UHPLC-ESI-MS method used for the measurement of MDAA containing samples are shown in Table S13.

**Table S13.** Validation parameters of the UHPLC-ESI-MS quantitation method of the bidirectional permeability in Calu-3 cell assay samples.

|  | **sanggenon C (SC)** | **sanggenon D (SD)** |
| --- | --- | --- |
| **Low concentration range calibration standards** | | |
| levels used | 8 | 6 |
| linear range [ng/mL] | 3.13–200 | 3.13–75.0 |
| R^2^ | 0.9999 | 0.9989 |
| LOD [ng/mL] | 3.13 | 3.13 |
| LOQ [ng/mL] | 6.25 | 6.25 |
| **High concentration range calibration standards** | | |
| levels used | 8 | 7 |
| linear range [ng/mL] | 19.5–2500 | 19.5–1250 |
| R^2^ | 0.9992 | 0.9980 |
| LOD [ng/mL] | 9.8 | 9.8 |
| LOQ [ng/mL] | 19.5 | 19.5 |

Each permeability sample was analysed in n = 6 replicates (except MA60 in apical compartment, direction A-B at t = 2h with n = 5 replicates) with 2 replicates on 1 well plate. Thus, the 6 replicates were derived from 3 different passage numbers of Calu-3 cells.

- 1. **Results of the pH-dependent solubility profile**

Results of the pH-dependent solubility profile of pure compounds SGC and SGD and both compounds within extracts MA 21 and MA60 are summarised in Table S14.

**Table S14.** Solubility of sanggenon C (SGC) and sanggenon D (SGD) as isolated stereoisomers and within the two multicomponent extract mixtures, MA21 and MA60, in four different buffer solutions with pH values of 1.2, 4.5, 6.8 and 7.4.

| Matrix | Compound | pH 1.2 solubility (µg/mL) | | pH 4.5 solubility (µg/mL) | | pH 6.8 solubility (µg/mL) | | pH 7.4 solubility (µg/mL) | |
| --- | --- | --- | --- | --- | --- | --- | --- | --- | --- |
| isolated | SGC | 4.47 | ± 1.43 | 8.53 | ± 3.66 | 18.5 | ± 6.2 | 47.1 | ± 15.7 |
| MA21 | SGC | < LOQ | - | < LOQ | - | 4.05 | ± 1.38 | 3.24 | ± 1.21 |
| MA60 | SGC | 6.88 | ± 9.70 | < LOQ | - | 12.7 | ± 6.3 | 13.6 | ± 4.1 |
|  |  |  |  |  |  |  |  |  |  |
| isolated | SGD | 111 | ± 18 | 121 | ± 17 | 304 | ± 108 | 493 | ± 161 |
| MA21 | SGD | < LOQ | - | < LOQ | - | < LOQ | - | < LOQ | - |
| MA60 | SGD | 20.6 | ± 14.6 | 19.7 | ± 1.3 | 56.8 | ± 18.9 | 94.9 | ± 9.5 |

1. **Permeability control experiments**
   1. Materials and Method

To better understand the cause of the one-directional TEER decrease observed when 20 µg/mL SGD samples were tested for permeation from the basolateral to apical direction, further studies were conducted to investigate this effect and its impact on the results. The experimental setup was as follows: SGC and SGD were added to either the basolateral or apical chambers of the wells or inserts. This time, fluorescein at a concentration of 40 µg/mL was always added to the apical chamber. Fluorescein was included because it is a non-permeability marker, and any slight influence on monolayer integrity would be detected by its rapid permeation through non-intact monolayers to the acceptor chamber. Inserts with and without cells, with the addition of just fluorescein, served as controls. Furthermore, MTT assays were conducted directly on the cell monolayer following the permeability studies to evaluate if cytotoxic processes were present.

The experiment was conducted with two different batches of Calu-3 cells, each from a different LOT, to determine whether the observed phenomenon was general or specific to a particular batch or passage number range. Calu-3 cells from batch 1, also used in the MDAA permeability studies (2.7), were from passages 21-22, while cells from batch 2 had a passage number of P12.

The experiment was initiated as described in section 2.7. Calu-3 cells were seeded on PET inserts (1.1 cm², 0.4 µm; Sarstedt, Nümbrecht, Germany) at a density of 1 x 10^5^ cells/insert, and placed in 12-well plates (VWR Chemicals, Radnor, USA). The cells were cultured for two weeks in a liquid-liquid interface to form an intact monolayer with tight junctions, which was assessed by measuring TEER values. Before testing the permeability of fluorescein in the presence of SGC/SGD, the medium was replaced with HBSS, and after a 30-minute incubation, TEER values were measured. Fluorescein permeability (donor concentration 40 µg/mL) was measured in the presence of 20 µg/mL of SGC or SGD in a bidirectional setup. For cell integrity assessment in the apical to basolateral direction (A-B), 520 µL of test solutions (SGC/SGD: 20 µg/mL; fluorescein 40 µg/mL) were added to the apical compartment, and 1700 µL of buffer was added to the basolateral chamber. For studies from the basolateral to the apical chamber (B-A), 520 µL of 40 µg/mL fluorescein in HBSS buffer was added to the apical compartment, and 1700 µL of the same SGC/SGD solutions were added to the basolateral compartment. Samples of 20 µL were withdrawn from the apical chamber at two time points: immediately after the addition of both solutions (t = 0 h) and after 120 minutes (t = 2 h). The donor samples were diluted with 180 µL of HBSS. From the basolateral chambers, samples of 200 µL were taken at the same time points. The plates were then placed on a shaking platform at 37 ± 0.37°C and 200 rpm for 2 hours. After incubation, the cells were washed, and the solutions were replaced with HBSS. After a further 30-minute incubation, TEER values were measured again to assess cell integrity. Samples taken at t_0h_ and t_2h_ were pipetted into a 96-well plate, and absorbance was measured at 490 nm using an EPOCH2 spectrophotometer (BioTek Instruments, Inc., Vermont, USA). A calibration curve with fluorescein, ranging from 20 to 0.5 mg/mL, was created to calculate the fluorescein concentration in the samples.

To assess potential cytotoxic effects on the monolayer, cell culture medium (10% FBS, 1% P/S) containing 0.45 mg/mL MTT reagent was added to the inserts (300 µL) and wells (1500 µL). Cells treated with fluorescein (40 µg/mL) served as a control. Samples were then incubated for 3.5 hours at 37°C. Following incubation, the MTT solution was removed, and DMSO was added to dissolve the formazan crystals (300 µL for inserts; 1500 µL for wells). The plates were incubated on a shaking platform at 200 rpm in the absence of light for 30 minutes. Subsequently, 100 µL samples from each well were pipetted into a 96-well plate, and the absorbance was measured at 570 nm using an Epoch 2 spectrophotometer.

- 1. Results and Discussion

The comparison of TEER values revealed significant differences in behaviour between the batches (**Figure S1 A-B**). While batch 2 exhibited higher overall TEER values compared to batch 1, a greater decrease in TEER was observed for batch 2 during the experiment, ultimately resulting in approximately the same TEER value after the experiment. Interestingly, the decrease in TEER observed in batch 1 during the measurement of the B-A transport of SGD was not observed in batch 2, indicating that this effect may be specific to the cell batch. In the MDAA permeability studies (**Figure 5**), the applied batch 1 exhibited a significant decrease in TEER values when SGD was directed from the basolateral to the apical side (B-A). This pronounced decline in TEER values was observed both in the previous and current experiments with batch 1 and not batch 2. Additionally, a decrease in TEER values was also observed with SGC in the B-A direction, although it was not as substantial as the decrease caused by SGD B-A.

To determine whether the observed decline in TEER influenced the permeability results, we recreated the experiments and added fluorescein, a non-permeability marker, to the cells. In an intact Calu-3 monolayer, no fluorescein permeation occurs to the other chamber. The experimental setup compared fluorescein permeation on inserts without a cell monolayer to that on a Calu-3 monolayer, as well as on a Calu-3 monolayer in the presence of SGC or SGD, in both transport directions. Particular focus was given to those conditions experiencing a reduction in TEER. However, no fluorescein permeation was detected in the transport studies involving a Calu-3 monolayer (LOQ 0.646 µg/mL; LOD 0.213 µg/mL; **Figure S1 C**). Fluorescein permeation only occurred through the membranes of inserts without a cell monolayer. None of the other tested substances affected the integrity of the Calu-3 monolayer to the extent that fluorescein permeability increased. This indicates that, despite the observed decline in TEER of the Calu-3 monolayer, there was no impact on the permeability assessments. Additionally, the remeasurement of TEER in the B-A directed SGD-test wells, after 24 hours of incubation with medium at 37°C, showed a recovery in TEER, suggesting that the TEER decrease is only a temporary effect.

Subsequently, a MTT assay was conducted to evaluate whether cytotoxic effects occurred on the Calu-3 monolayers, particularly to ascertain if the B-A directed TEER reduction caused by SGD was due to cell-damaging processes. Neither batch 1 nor batch 2 showed a decrease in cell viability after the permeability studies, implying that the TEER decline is not caused by damaged cells (**Figure S1 D**).


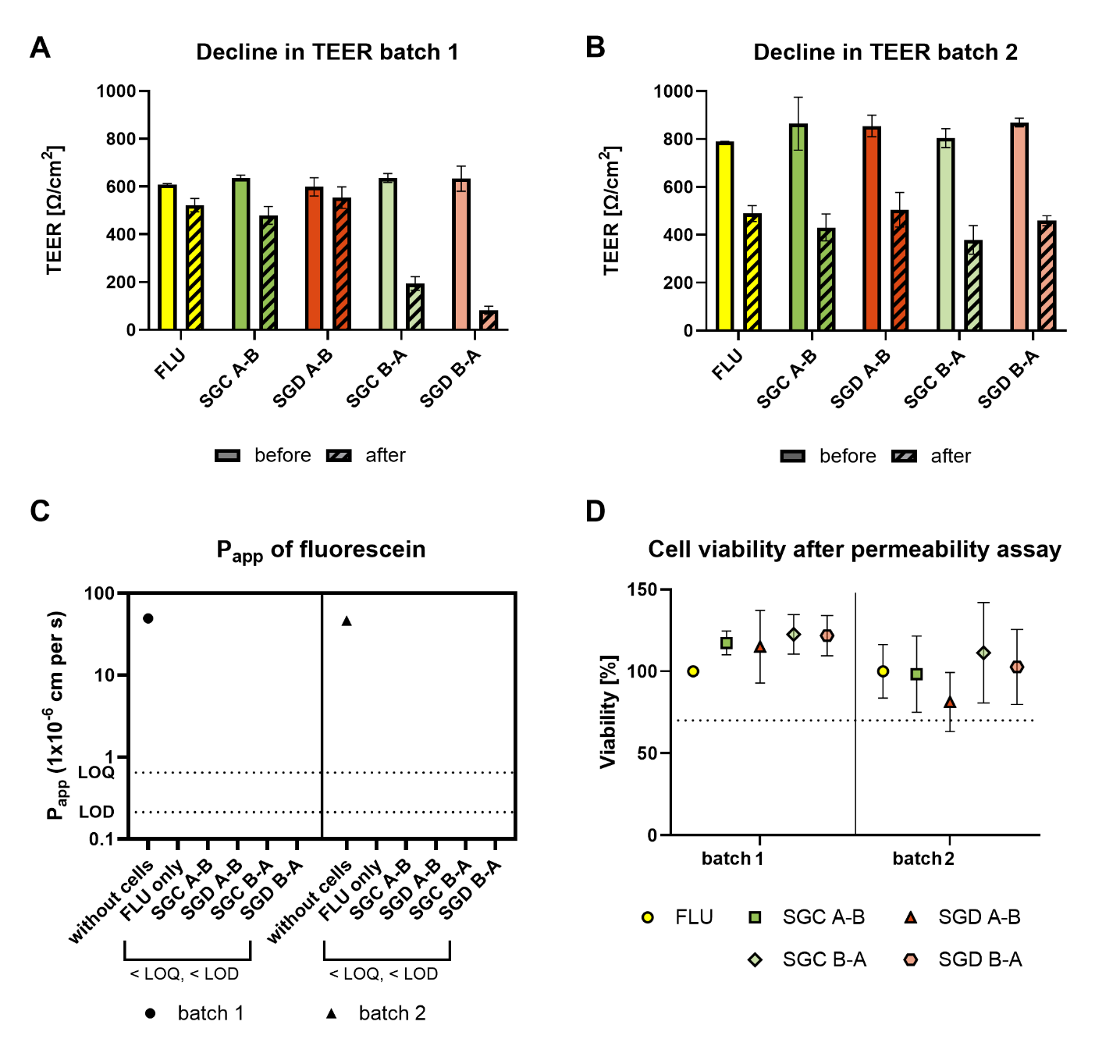


**Figure S2.** A) Decline in TEER values of Calu-3 cell batch 1 before and after the permeability experiment. B) Decline in TEER values of Calu-3 cell batch 2 before and after the permeability experiment. C) Comparison of apparent permeability (Papp) values of fluorescein on inserts without a cell monolayer versus on a Calu-3 monolayer, in the presence of fluorescein alone or in a bidirectional setup with SGC or SGD. D) Cell viability assessed for both batches of cells after the permeability studies with the Calu-3 monolayer. Reduction under 70 % of viability was considered as cytotoxic (dotted line).

In conclusion, the observed one-directional decrease in TEER caused by SGD appears to be a cell-batch specific phenomenon with an unknown molecular mechanism. Importantly, despite the decline in epithelial resistance, there was no significant increase in permeability, indicating that the robustness of the permeability results remains unaffected.

1. **Results of uptake in Calu-3 cells**

Results of the uptake experiment via flow-injection time-of-flight mass spectrometry (FIA-TOFMS) in negative ion mode for ion m/z 707.2127 with validation parameters of 3.14E+05 for slope, 6.58E+04 for intercept and 0.9840 for R^2^ are listed in Table S15.

**Table S15.** Overview of the measurement data of the uptake in Calu-3 cells. Negative concentration values observed in vehicle-treated cell extracts are due to numerical errors close to the detection limit (e.g. ion intensities lower than the intercept of the regression model).

| Treatment | Treatment concentration | Time  [hours] | Ion intensity  [counts] | | | Concentration  [pg/µL] | | | Cell number  [cells/µL] | Normalized concentration  [pg/cell] | | | Mean  [pg/cell] | SD  [pg/cell] |
| --- | --- | --- | --- | --- | --- | --- | --- | --- | --- | --- | --- | --- | --- | --- |
|  |  |  | 1 | 2 | 3 | 1 | 2 | 3 |  | 1 | 2 | 3 |  |  |
| Sanggenon C | 50 µg/mL | 4 | 1.41E+06 | 1.28E+06 | 1.29E+06 | 4284 | 3851 | 3902 | 2050 | 2.09 | 1.88 | 1.90 | 1.96 | 0.12 |
| Sanggenon C | 50 µg/mL | 12 | 4.35E+06 | 4.28E+06 | 3.92E+06 | 13620 | 13397 | 12248 | 2362 | 5.77 | 5.67 | 5.19 | 5.54 | 0.31 |
| Sanggenon C | 50 µg/mL | 24 | 5.70E+06 | 5.37E+06 | 5.54E+06 | 17926 | 16862 | 17411 | 2148 | 8.34 | 7.85 | 8.11 | 8.10 | 0.25 |
|  |  |  |  |  |  |  |  |  |  |  |  |  |  |  |
| Sanggenon D | 50 µg/mL | 4 | 2.07E+05 | 1.54E+05 | 4.30E+05 | 449 | 281 | 1158 | 1946 | 0.23 | 0.14 | 0.60 | 0.32 | 0.24 |
| Sanggenon D | 50 µg/mL | 12 | 4.23E+05 | 4.17E+05 | 3.85E+05 | 1137 | 1116 | 1014 | 2014 | 0.57 | 0.55 | 0.50 | 0.54 | 0.03 |
| Sanggenon D | 50 µg/mL | 24 | 5.54E+05 | 7.59E+05 | 5.91E+05 | 1552 | 2206 | 1671 | 2638 | 0.59 | 0.84 | 0.63 | 0.69 | 0.13 |
|  |  |  |  |  |  |  |  |  |  |  |  |  |  |  |
| Vehicle (DMSO) | 0.25% | 4 | 1.89E+04 | 2.87E+04 | 4.97E+04 | -149 | -118 | -51 | 2689 | -0.06 | -0.04 | -0.02 | -0.04 | 0.02 |
| Vehicle (DMSO) | 0.25% | 12 | 3.73E+04 | 2.03E+05 | 8.11E+04 | -91 | 436 | 49 | 2316 | -0.04 | 0.19 | 0.02 | 0.06 | 0.12 |
| Vehicle (DMSO) | 0.25% | 24 | 1.45E+04 | 1.95E+05 | 1.92E+04 | -163 | 411 | -148 | 2513 | -0.07 | 0.16 | -0.06 | 0.01 | 0.13 |
|  |  |  |  |  |  |  |  |  |  |  |  |  |  |  |
